# Animal biosecurity framework development, implementation and evaluation in a veterinary education establishment

**DOI:** 10.1080/01652176.2026.2626257

**Published:** 2026-02-05

**Authors:** Claude Saegerman, Constance Wielick, Véronique Renault, Priscilla Burnotte, Christine Grignet, Laurent Leinartz, Maxime Harmegnies, Sophie Tasnier, Christiaen Remy, Calixte Bayrou, Nicolas Ochelen, Tatiana Art, Pierre Lekeux, Jason W. Stull, Marie-France Humblet

**Affiliations:** a Department of Infectious Diseases, Research Unit in Epidemiology, Risk Analysis and Biosecurity applied to Veterinary Sciences, University of Liège, Liège, Belgium; b Faculty Biosecurity Unit, University of Liège, Liège, Belgium; c Council Member of the World Animal Biosecurity Association and Management Committee member of the COST Action Biosecurity Enhanced Through Training Evaluation and Raising Awareness (BETTER) CA20103;; d Department of Small Animal Clinical Science, Faculty of Veterinary Medicine, University of Liège, Liège, Belgium; e Department of Occupational Protection and Hygiene, Unit Biosafety, Biosecurity and Environmental Licences, University of Liège, Liège, Belgium; f ToolBox, CARE VetMeDiSim, Faculty of Veterinary Medicine, University of Liège, Liège, Belgium; g Clinical Department of Production Animals, Faculty of Veterinary Medicine, University of Liège, Liège, Belgium; h Administrative Office - Logistics Unit, Faculty of Veterinary Medicine, University of Liège, Liège, Belgium; i Department of Functional Sciences, Physiology and Sport Medicine, Faculty of Veterinary Medicine, University of Liège, Liège, Belgium; j Dean of the Faculty of Veterinary Medicine, University of Liège, Liège, Belgium; k European Association of Establishments for Veterinary Education (EAEVE), Vienna, Austria; l Atlantic Veterinary College, University of Prince Edward Island, Charlottetown, Canada

**Keywords:** Animal, biosecurity, veterinary education establishment (VEE), standard operating procedure (SOP), framework, development, implementation, evaluation, accreditation

## Abstract

Over the last decades, biosecurity has received increasing attention in veterinary medicine and was recently integrated as a competency for One Health field epidemiology framework by international bodies. It is also a standard in the European System of Evaluation of Veterinary Training and in the accreditation of veterinary colleges by the American Veterinary Medical Association Council on Education. To help veterinary students and staff acquire biosecurity skills within veterinary education establishments, we first develop animal biosecurity research, and we spread its results through four interconnected instruments: biosecurity standard operating policies and procedures, a dedicated biosecurity website, an annual biosecurity day, and the production of checklists to assess the biosecurity level of compliance. The use of biosecurity standard operating procedures, the number of visits on the faculty biosecurity website, the number of people trained, and regular biosecurity audits performed are all factors that have contributed to the animal biosecurity to comply with the requirements of the European Association of Establishments for Veterinary Education and by the Council on Outcomes-based Veterinary Education, in the CBVE 2.0 book published by the American Association of Veterinary Medical Colleges. These approaches also contribute to the acquisition and maintenance of the accreditation delivered by the ad hoc bodies. The participation of students in the process allows a better comprehension and appropriation of animal biosecurity.

## Introduction

**Figure 1.**
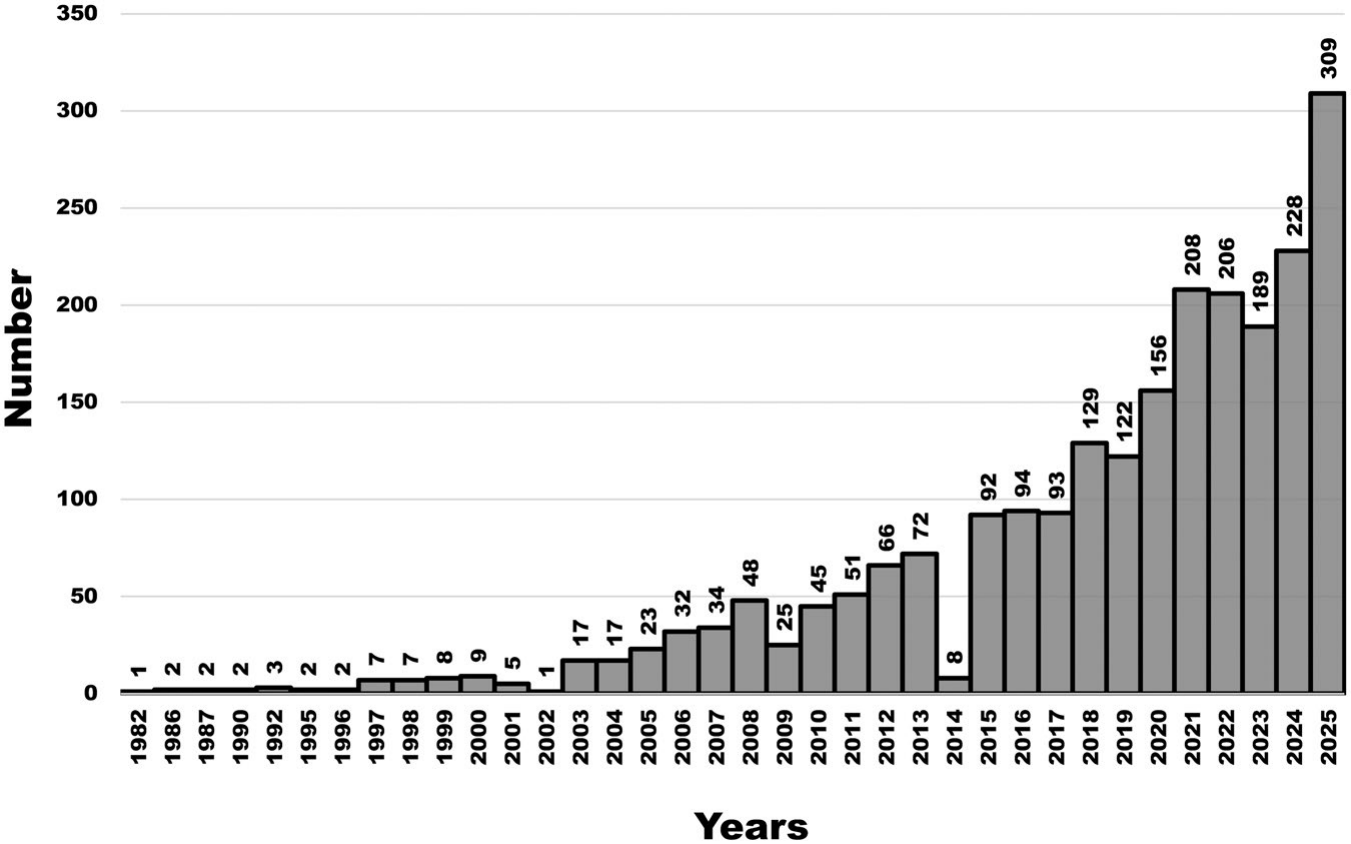
Number of publications (vertical axis) present in PubMed (US National Library of Medicine, National Institutes of Health) mentioning ‘biosecurity’ or ‘infection control’ and ‘animal’ in title/abstract, by year of publication (horizontal axis), 1992–2025 (*N* = 2315). Data were extracted on 31 December 2025.

Over the last decades, animal biosecurity has received increasing attention in veterinary medicine (Figure 1) and has been integrated into the Animal Health Law (European Parliament and Council 2016).

Several definitions of animal biosecurity exist, but according to a recent world survey, the most significant popular definition was the one that conceptualised the rules of 5Bio’s (bio-exclusion, biocontainment, bio-compartmentation, bio-prevention, and bio-preservation) (Saegerman et al. 2023). Within this definition, animal biosecurity consists in all measures (1) to limit the risk of introduction (bio-exclusion) into an animal facility; (2) to limit the spread of the pathogen within the same facility (bio-compartmentation); (3) to limit the spread of a pathogen outside the facility (inter-herd transmission) (bio-containment); (4) to prevent the risk of human contamination (bio-prevention); and (5) to prevent any environmental bio-contamination and persistence of the pathogen (bio-preservation) (Saegerman et al. 2023).

**Figure 2.**
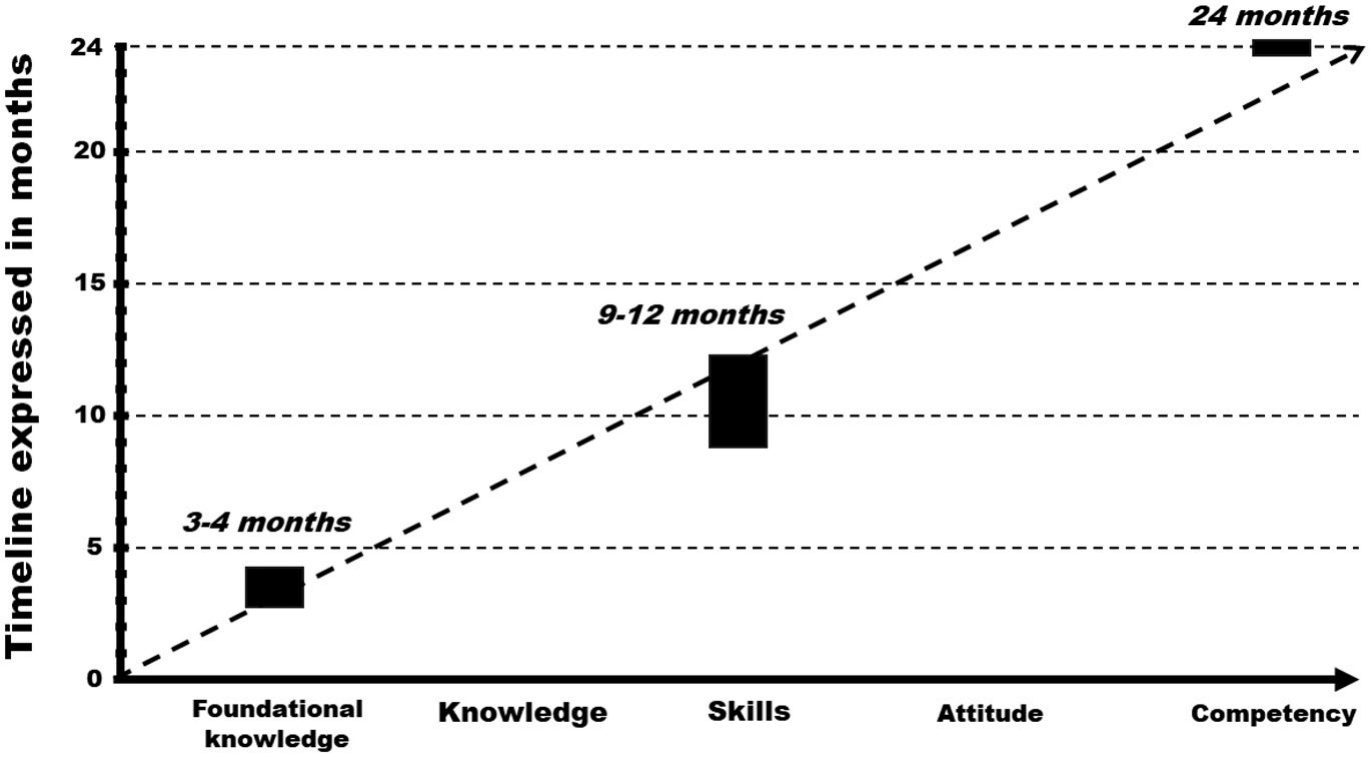
Training levels and timelines for acquiring knowledge, skills, attitudes and competencies in One Health field epidemiology training programmes (adapted from COHFE, 2024). *Legend.* Knowledge is the assimilation of information through learning. Skill is the ability to apply knowledge and to complete tasks and solve problems. Attitude is the person’s feelings, values and beliefs, which influence their behaviour and the performance of tasks. Competency is the proven ability to apply knowledge, skills and personal, social and methodological abilities (attitudes and behaviours), in work or study situations and in professional and personal development in terms of responsibility and autonomy (COHFE 2024).

In terms of education, biosecurity was recently integrated by WHO, FAO and WOAH as a competency for One Health field epidemiology framework, especially in domain number six related to infection prevention and control, biosafety and biosecurity (COHFE 2024); it includes three subdomains, i.e. preparedness, implementation procedures, and evaluation of continuous quality improvement (COHFE 2024). Training levels and timelines for acquiring biosecurity knowledge, skills, attitudes and competencies in One Health field epidemiology training programmes are described in Figure 2 (COHFE 2024).

The European Association of Establishments for Veterinary Education (EAEVE) monitors the harmonisation of the minimum standards set down in the study programme for veterinary surgeons in the European Union Directive 2005/36. This is implemented through the European System of Evaluation of Veterinary Training, which is managed by the EAEVE in collaboration with the Federation of Veterinarians of Europe (FVE). In America, the evaluation of veterinary training is monitored by the American Veterinary Medical Association (AVMA) - Council on Education (COE). In this work, we focus on the situation in Europe. One objective of the EAEVE is to maintain a list of Evaluated and Accredited Veterinary Education establishments (VEE). Other objectives are to reinforce cooperation between member establishments and to act as a discussion forum to improve and harmonize veterinary education. Additional tasks are the facilitation to exchange information, staff, student and teaching materials between members. Members are the faculties, schools and universities involved in teaching and research in veterinary medicine and science. In 2025, out of the 112 VEEs existing in Europe, 100 are members of the EAEVE; among them, 79 VEEs are accredited, 5 are with pending accreditation, 12 have not yet been visited and the remaining 4 are not accredited. Most of the non-member VEEs have not graduated their first cohort of students, which is a prerequisite. Some non-European VEEs are Associate Members of EAEVE, i.e. from Asia, the Middle East, North Africa and South America.

Biosecurity is a standard in the European System of Evaluation of Veterinary Training. Indeed, some EAEVE standards should be considered as related to biosecurity, especially standards 4.3 (livestock facilities, animal housing, core clinical teaching facilities and equipment used by the VEE for teaching purposes), 4.6 (isolation and containment of animals with communicable diseases) and 4.9 (biosecurity standard operating policies and procedures (SOPs) integrated in a manual, its dedicated Committee and a system of Quality assurance). A critical point considered by EAEVE experts during a visit is the availability of relevant biosecurity SOPs (manual), easily accessible on a website, amended regularly, taught to staff and students, posted on relevant places (colour code on the floor, pictograms or QR codes on the doors), and fully and correctly implemented (e.g. clean barrier, no cross-over between clean and dirty areas) in all relevant places (e.g. clinics, anatomy & necropsy rooms, isolation facilities, ambulatory clinics, teaching farms). In addition, veterinary education and practices need to be environmentally-conscious implementing the 3 R approach, i.e. reducing waste, reusing tools, and recycling resources and products (e.g. personal protective equipment, disposable materials, single-use plastics, water consumption, greenhouse gas emission) but with the same evidence-based level of protection.

To help veterinary students and staff acquire biosecurity skills, we first develop animal biosecurity research, and we spread its results through four interconnected instruments: biosecurity standard operating procedures, a dedicated biosecurity website, an annual biosecurity day, and the production of checklists to assess the biosecurity level of compliance. The aim of this paper is to present the developed biosecurity materials and experiences to contribute to the spread of biosecurity standards and evaluation tools in VEE, using a Veterinary Faculty as an example.

## Materials and methods

A VEE biosecurity unit was created at the Veterinary Medicine Faculty of the University of Liège (ULiège). This University includes eleven faculties representing the humanities, health sciences, science and technology, one of which is the Veterinary Medicine Faculty, in a separate building. The first task of the VEE biosecurity unit was to develop and refine biosecurity standards operating policies and procedures (SOPs). The second task was the creation of a biosecurity website for VEE students, staff and visitors. The third task was the organization of a VEE annual Biosecurity Day combining didactic presentations and practical workshops to popularize and spread scientific knowledge operationally among the VEE staff and students. The fourth task was to produce checklists to properly assess the level of biosecurity compliance towards SOPs. All these materials and methods were created based on existing documentation such as: the 2018 Infection Control, Prevention, and Biosecurity Guidelines elaborated by the American Animal Hospital Association (AAHA) (Stull et al. 2018), the 2023 standard operating procedure of the European System of Evaluation of Veterinary Training (ESEVT SOP 2023), the accreditation policies and procedures of the AVMA-COE (AVMA, 2025a), and the FAO Laboratory Mapping Tool (Mouillé et al. 2018); and other relevant supporting scientific literature helped developing the tools (Brass et al. 2017; Morley, 2002; Humblet et al. 2017; Saegerman and Humblet 2018; Sarrazin et al. 2018; Humblet et al. 2020a, 2020b, 2020c; Humblet and Saegerman 2023).

## Results

### Creation of a veterinary education establishment biosecurity unit

The first step to promote biosecurity was the creation of a specific VEE unit dedicated specifically to animal biosecurity (Box 1). This unit has an advisory capacity, targeting biosecurity within the framework of teaching activities involving a biological risk (clinics, anatomy and necropsy room, farms, paraclinics, teaching labs, practical activities and tutorial classes) and provides recommendations to the VEE. Advisements of the VEE biosecurity unit addresses the adoption of biosecurity procedures (SOPs) and adequation of infrastructures where live or dead animals, animal products and biological samples are found. The unit defines procedures allowing the assessment and management of biological risks within the framework of teaching activities, the assessment of compliance with biosecurity SOPs and the surveillance of antibiotic resistance in the VEE.

Box 1. Creation of a veterinary education establishment biosecurity unit.

**Table:** 

Define the members of the Executive Committee for daily activities (president of the unit and a biosecurity logistician) Obtain formal recognition of the VEE for the biosecurity team to be a consultative body Ensure all animal species and teaching laboratories are considered Define the tasks of the biosecurity team and have them validated by the VEE Organise regular meetings (e.g. at least 3 per year, with additional sessions if needed) Provide feedback through meetings, meeting reports and an annual report

*Legend*. VEE: Veterinary Educational Establishments.

The main missions of the VEE biosecurity unit are: (i) the development and the update of the biosecurity SOPs/Manual and website, with special focus on new legislations, emergence of infectious diseases and recommendations from bodies, either internal to the University, such as the Department of Occupational Protection and Hygiene (SUPHT), or external like the Department of Prevention and Occupational Health Medicine (COHEZIO), and international bodies that delivered biosecurity standards like the COHFE at the world level, and the EAEVE at the European level; (ii) the implementation of a biosecurity educational programme for all actors of the VEE through, among others, the organisation of the annual Biosecurity Day especially dedicated to the staff members of the VEE but also for interested veterinary practitioners; (iii) the assessment of human and logistical means required to reach the objectives mentioned above, in collaboration with the relevant departments of the VEE (strategic plan) and (iv) the elaboration of crisis scenarios. An activity report is produced annually that summarises all activities performed by the VEE biosecurity unit and its executive committee. Each annual activity report is shared with all members of the VEE biosecurity unit and invited members; it is uploaded on the biosecurity website for all interested parties (transparency).

**Figure 3.**
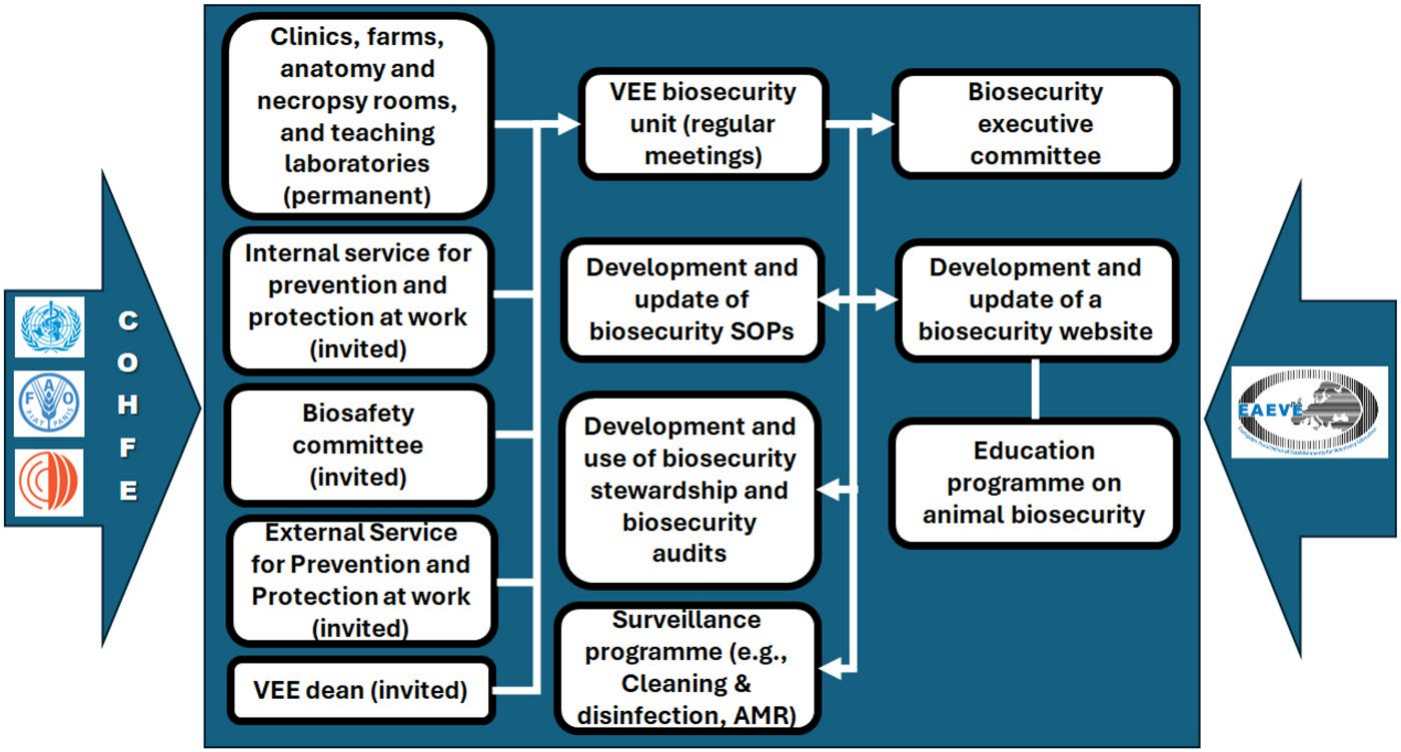
Organisation and governance of VEE biosecurity. *Legend.* COHFE, the Competencies for One Health field epidemiology framework proposed by WHO-FAO-WOAH; SOPs, standard operating policies and procedures; VEE, Veterinary Education Establishment; AMR, antimicrobial resistance; EAEVE, European Association of Establishments for Veterinary Education.

Regarding the composition of the VEE biosecurity unit, members are assigned by the VEE Council for a 2-year term, renewable mandate. The President is elected internally, for a 2-year- and renewable mandate as well. Each VEE Department is represented in the VEE biosecurity unit; some preclinical and clinical students are also members of the body. Permanent guests include: the VEE Biosafety Officer, an Occupational Health Doctor, the President of the VEE Biosafety Committee (responsible for activities related with contained use of Genetically Modified Microorganisms (GMMs) and pathogens, i.e. mainly research laboratories and laboratory animal facilities), and the VEE Dean (Figure 3). This guest status may facilitate the implementation of certain proposals from the VEE biosafety unit, which provides advisory guidance.

Regarding the operation of the VEE biosecurity unit, meetings are held at least three times a year, and whenever circumstances require, to address ongoing matters and assess submitted issues. A report is systematically written by the secretary, validated by all members of the VEE biosecurity unit and forwarded to the Dean, the Dean’s office and any person concerned by topics discussed.

### Elaboration of biosecurity standard operating policies and procedures integrated into a manual

The manual of biosecurity SOPs constitutes the first biosecurity instrument for a VEE. This manual is subdivided into eighteen chapters (Table 1 and Appendix A) and is updated every 5 years or sooner if warranted. These SOPs are now widely used in European VEEs and worldwide. The last update was made in June 2025.

**Table 1.** Table of contents of the biosecurity standard operating policies and procedures integrated into a manual (example of the Faculty of Veterinary Medicine, University of Liège, Belgium).

Chapter 1	General Biosecurity SOP
Chapter 2	Equine Biosecurity SOP
Chapter 3	Ruminant Biosecurity SOP
Chapter 4	Pig Farm Biosecurity SOP
Chapter 5	Small Animal Biosecurity SOP
Chapter 6	Bird, Pet Rabbit / Rodent / Pet Poultry, Zoological and Exotic Animal (BRRPZE) Biosecurity SOP
Chapter 7	Food Science Biosecurity: Extra Muros Practical Works
Chapter 8	Experimental Farm (FePEx) Biosecurity SOP
Chapter 9	Veterinary Management of Animal Resources (DRA) Biosecurity SOP
Chapter 10	Anatomy Biosecurity SOP
Chapter 11	Wildlife Health and Pathology Biosecurity SOP
Chapter 12	Teaching Laboratories and Diagnostic Biosecurity SOP
Chapter 13	Pest control SOP
Chapter 14	Laundry SOP
Chapter 15	Antimicrobial Resistance SOP
Chapter 16	Quality Assurance and Biosecurity in the Faculty of Veterinary Medicine
Chapter 17	Crisis Scenarios SOP
Chapter 18	Future Tasks of the Biosecurity Unit (CFB)
	References
	Appendices

*Legend.* SOP, standard operating policy and procedure; DRA, Department of Animal Resources.

### Development of a dedicated biosecurity website

**Figure 4.**
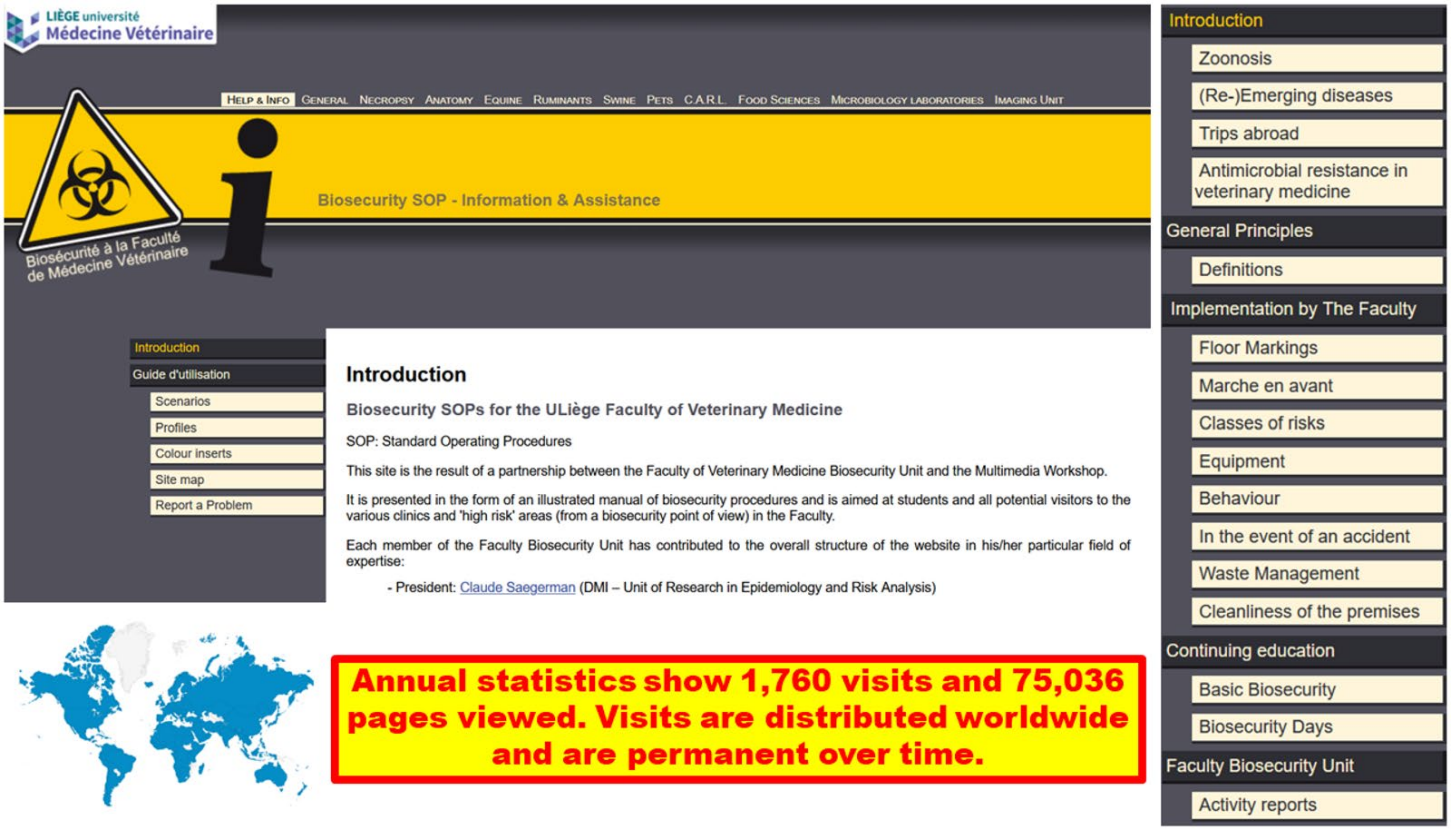
The biosecurity website is a concise, operational and educational translation of the veterinary education establishment biosecurity standard operating policies and procedures. *Legend.* On the left, the welcome page of the website; on the right, an example of items for the general information.

Since 2012, a website dedicated to biosecurity (https://www.fmv-biosecurite.ulg.ac.be/?langue=en) was developed and updated by the VEE biosecurity unit and the professor in charge of teaching biosecurity in the VEE (Figure 4). This website constitutes the second instrument and translates the biosecurity SOPs (manual) into concise, operational (practical) educational information with extensive iconography. Key stakeholders can visit this website (i.e. students or visitors, private veterinarians, staff, students with reduced mobility). The first page, called ‘help and information’, contains an introductive section explaining the importance of biosecurity in VEE and a user guide that includes several scenarios (e.g. classification of animals according to their category of risk in different clinics), profiles (content adapted to each user profile), colour inserts (blue inserts highlight the fact that the content is not directly relevant to a specific profile of the current user and sepia inserts highlight the fact that the content has already been set out elsewhere, e.g. in another scenario), site map (detailed table of the website contents), and a problem report (see Section “Reporting a biosecurity issue”). The other pages are directly related to the different chapters of the Manual of biosecurity SOPs (e.g. ‘pets’ correspond to the biosecurity SOPs for the small animal clinic). Annual website statistics show a mean of 1760 visits and 75,036 pages viewed per year. Visits are distributed worldwide and are consistent over time.

### Biosecurity teaching aligned with standard operating procedures

#### Course in the preclinical degree in veterinary medicine

The same biosecurity module is delivered in different VEEs. According to COHFE, biosecurity is considered as a core competency within the One Health field epidemiology framework. Accordingly, a biosecurity component included in the course ‘Veterinary Epidemiology, risk analysis, biosecurity and good veterinary practices’ is taught at ULiège, UNamur and UCLouvain in the third year of the preclinical degree in veterinary medicine. This course is organised as follows: a 2-hour theoretical introduction covering the main principles of biosecurity and selected topics on biosecurity quality assurance, followed by 30 h of e-learning, *via* the ULiège eCampus platform and the companion website specifically dedicated to biosecurity in the VEE (see Section “Development of a dedicated biosecurity website”). Didactic materials include lecture slides (theoretical course), biosecurity SOPs and the content of the biosecurity website. Each chapter is related to a SOP and is followed by a formative self-evaluation. Student must correctly answer a short multiple-choice questionnaire after each chapter to unlock the access to the next chapter. Each student of the ULiège, ULouvain and UNamur needs to pass a final written examination, which consists of a multiple-choice questionnaire. It would be desirable to harmonise the biosecurity component of veterinary epidemiology across the various universities offering preclinical courses to ensure that all students are prepared before embarking on clinical courses, which are only offered by ULiège and UGent in Belgium.

#### Paraclinical activities on biosecurity

**Figure 5.**
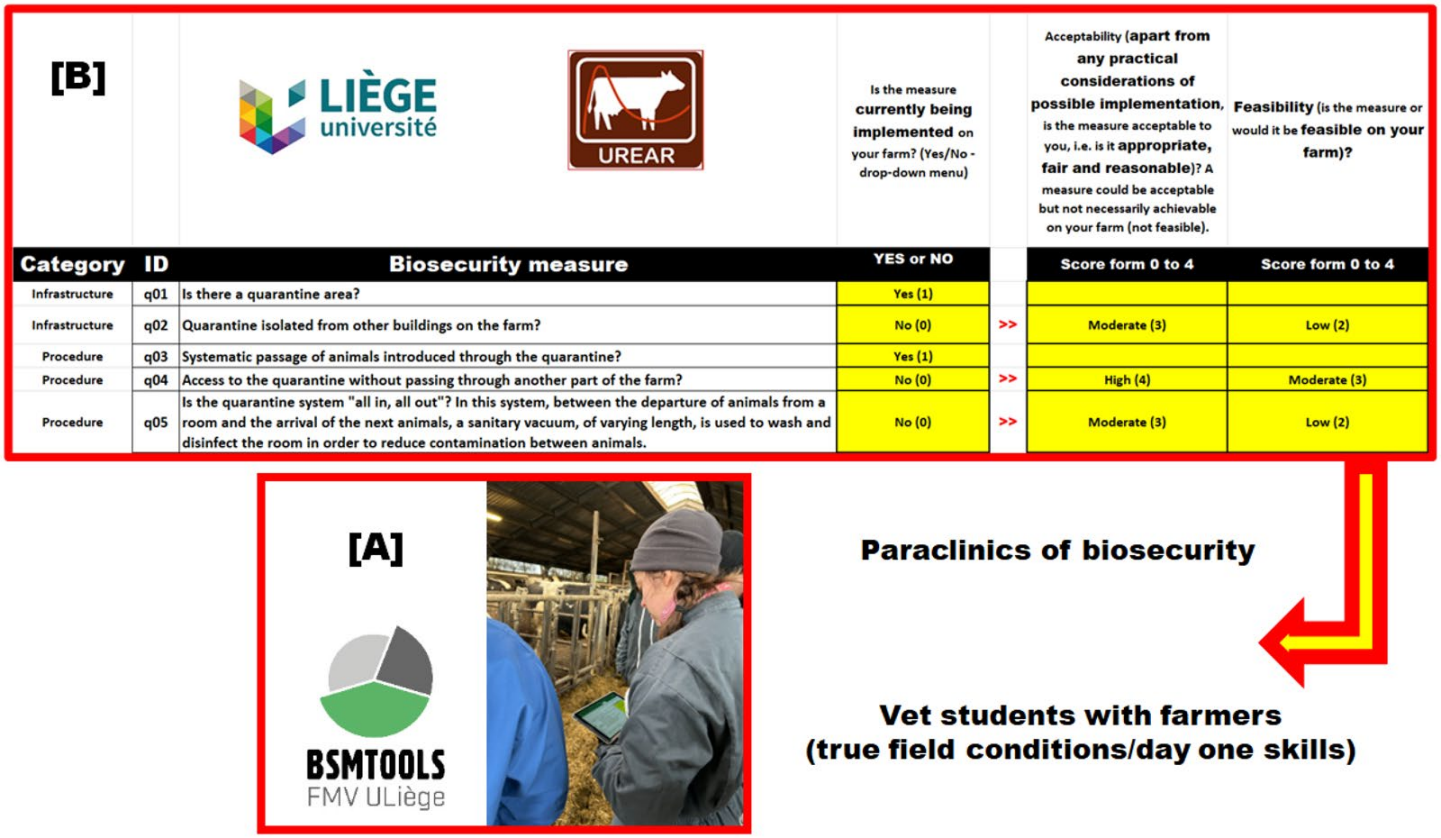
Each digital tool called BSMTOOLS [A] is used by students in field conditions to conduct a biosecurity audit [B] for a specific disease (paraclinics). *Legend.* The audit aims to assess the level of protection on a farm and identify biosecurity measures (BSM) that could be implemented to improve the level of protection against sources of introduction of a specific disease (e.g. influenza). To answer this question, the average situation on the farm over the year is considered. The student must complete the entire questionnaire with the farmer, cross-checking the answers with what they observed during the preliminary visit to the farm (cross-check validation).

Biosecurity paraclinical activities are organized in the second year of the clinical degree in veterinary medicine. Note that paraclinics are defined as specialized activities that support diagnosis, research, or education without being directly involved in the clinical care of animals. They include preparation and the conduct of a biosecurity audit by students, using digital tools (*via* tablets) in a farm, followed by veterinary practice simulations (2 x ½ day) (Figure 5).

#### Continuing education

Biosecurity training sessions for technical staff and stablemen are conducted annually. All personnel working in areas hosting teaching activities are required to participate. The training is mandatory, provided under the authority of the management to ensure the compliance with the chain of command, and its content is validated by the VEE biosecurity unit.

In addition, students in clinical degree must complete a several-month internship in veterinary structure (private veterinarians, centres, clinics) or within various institutions or companies. Therefore, educating private veterinarians on animal biosecurity (continuing education) is crucial to maintain a high level of biosecurity in all places where animals are kept whatever the structure and to set an example for students in a real-world context. Indeed, continuing education is delivered *via* the annual biosecurity day (see Section “Annual biosecurity day”) and *via* conferences. Conferences target veterinary practitioners, for example, through one-day training sessions on biosecurity and antimicrobial resistance in a One Health framework (25 November 2024, FORMAVET). Continuing education activities are also organized for VEE representatives, such as an invited lecture on the development, implementation, and evaluation of biosecurity SOPs during the 38th EAEVE General Assembly and Educational Day (Dublin, 12–13 June 2025). In addition, invited talks will address biosecurity challenges related to African Swine Fever for members of the Swine and Poultry Infectious Diseases Research Center (Saint-Hyacinthe, Canada; 4 October 2024). Training activities have also been provided for members of the COST Action CA20103 BETTER, including a four-day training school at ULiège covering the design, implementation, and assessment of biosecurity SOPs in a VEE (14–18 November 2022).

Continuing education is also supported by contributions to book chapters on animal biosecurity (Sarrazin et al. 2018; Saegerman and Humblet 2018), publications on biosecurity compliance (Humblet et al. 2017; Humblet and Saegerman 2023) and integrated management of blood-feeding arthropods (Humblet et al. 2020a, 2020b, 2020c). It is further reinforced through research projects. Teaching and research combined offer significant added value over teaching alone by bringing cutting-edge knowledge, fostering deeper student engagement with current advancements, providing richer mentorship, and enhancing faculty skills, leading to a more dynamic, relevant, and prestigious academic environment, though balancing them effectively remains a challenge.

In addition, several final-year student theses have been undertaken to promote the appropriation of biosecurity among students. One example of a thesis topic includes the implementation of a quality approach regarding biosecurity within the ULiège Faculty of Veterinary Medicine. Other topics address how to assess the biosecurity level in stud farms, based on a survey among 70 Belgian and French breeders. Additional work focuses on reducing the incidence of nosocomial infections in the intensive care unit of a small animal hospital. One thesis proposes an action plan against African swine fever in the pig farm of the ULiège Faculty of Veterinary Medicine. Another explores the viruses carried by European bats and presents a method for prioritizing zoonotic and epidemic risks in France and Belgium.

### Annual biosecurity day

Since 2013, an annual biosecurity day is organised by the VEE biosecurity unit and the Internal service for prevention and protection at work (SUPHT). This biosecurity day combines morning theoretical presentations and afternoon practical workshops (practical exercises, world café, or case studies to solve) to popularise and disseminate operational scientific knowledge among VEE staff and students. Each biosecurity day is primarily aimed at the VEE staff, within the frameworks of continuing education, but also welcomes students and private practitioners, authorities, and other interested stakeholders. This education day is free for all ULiège staff members and students, with opportunities for interactions and discussions between all participants and trainers (e.g. coffee break, lunch). The day is certified as continuing education by the Federal Authority. Topics differ every year and focus on practical biosecurity as implemented in the sectors of the VEE housing teaching activities, mainly clinics and microbiology laboratories. Topics are selected by the VEE biosecurity unit based on suggestions from participants to the previous edition, to address as much as possible their expectations and needs (Table 2). For example, the 2025 edition focused on cleaning and disinfection in veterinary medicine. The VEE staff members are required to attend at least once every five years (i.e. continuing education). To date, 13 editions have been organised; they gathered around 100 participants each year (ranging from 90 to 140). All presentations are made available on the biosecurity website.

**Table 2.** Topics of the VEE biosecurity days since 2013.

Year	Topic	Number of participants
2013	Biosecurity measures to take upon returning from a trip abroad. Managing antibiotic resistance at a VEE	71
2014	Practical aspects of controlling insects that carry pathogens	90
2015	Importance of biosecurity in managing the risk of introduction of exotic diseases: canine leishmaniasis and heartworm disease, African swine fever, foot-and-mouth disease, and West Nile fever	65
2016	Basic hygiene and biosecurity in clinics	117
2017	Management of infectious patients	119
2018	Importance of biosecurity in crisis management	86
2019	Multitopic day	143
2020	Field biosecurity	108
2021	VEE initiatives related to COVID-19 and biosecurity	100
2022	Zoonotic risks associated with disaster situations – focus on floods	112
2023	Influenza viruses	66
2024	The role of veterinarians in the prevention of zoonotic diseases	70
2025	Cleaning and disinfection in veterinary medicine	80

*Legend.* VEE: Veterinary Education Establishment.

### Implementation and auditing of biosecurity standard operating policies and procedures

**Figure 6.**
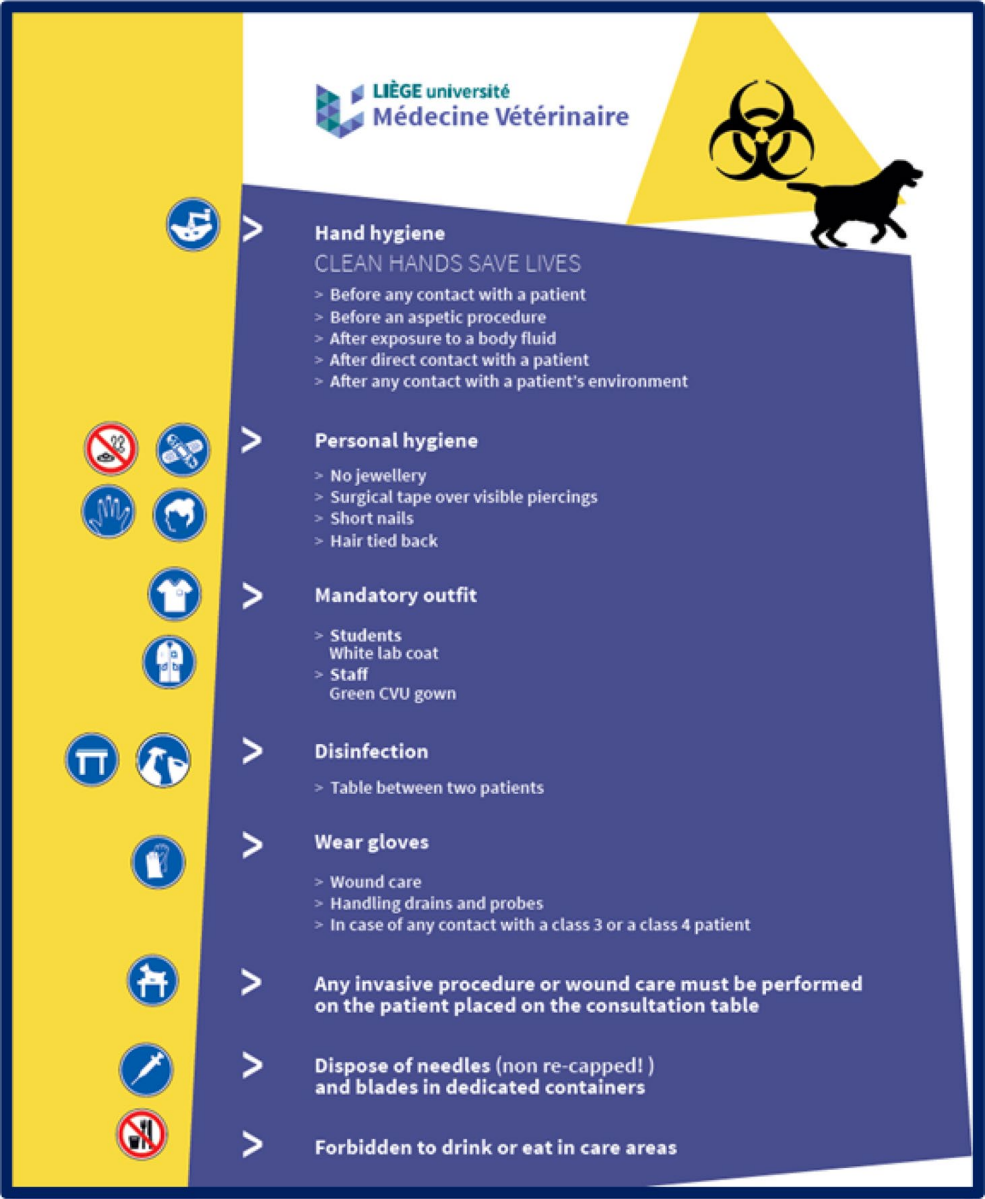
Example of a panel in the small animal clinic highlighting top biosecurity measures.

Implementing biosecurity Standard Operating Policies and Procedures (SOPs) involves establishing and enforcing measures to prevent the introduction and spread of harmful organisms such as pathogens, pests, and invasive species. These SOPs typically include isolation, cleaning, and disinfection protocols, as well as risk assessments and regular monitoring. To support the implementation of biosecurity measures within the VEE, panels are displayed on walls of different clinics and laboratories used in teaching activities. These panels highlight the top 10 biosecurity measures that all people, mainly students and staff, must follow during their activities in the facilities (Figure 6). These measures were defined by the heads of clinics, laboratories and activities, in collaboration with the VEE biosecurity unit.

A system of signposting is implemented on doors and walls throughout the VEE facilities. Such a system highlights key measures and behaviours, informs people about specific hazards (biological, physical and chemical) and indicates the mandatory personal protective equipment. Biosecurity signposting is the responsibility of the heads of clinics and laboratories, under the supervision of the VEE biosecurity unit. For standardisation, biosecurity signs are consistent across all VEE sites and were developed in collaboration with the ULiège Department of Infrastructure and the SUPHT. Safety signs (e.g. localisation of emergency exits, fire extinguishers, etc.) comply with current legislation and are the responsibility of the SUPHT.

Quality assurance in biosecurity is the responsibility of the VEE biosecurity unit. The unit works as follow: clinics and sectors exposed to biological risks linked to teaching activities submit any biosecurity-related issue to the unit. With the help of the biosecurity logistician, the unit gathers information to build a case, which is then discussed during meetings. Proposed solutions are communicated to the applicant as formal recommendations. As an example, the small animal clinic consulted the unit regarding a series of surgical site infections. The unit proposed an action plan consisting of 1) an audit of practices, 2) environmental sampling to validate the cleaning and disinfection protocols and 3) suggestions of improvements based on the audit and the sampling results, following an evidence-based approach.

**Figure 7.**
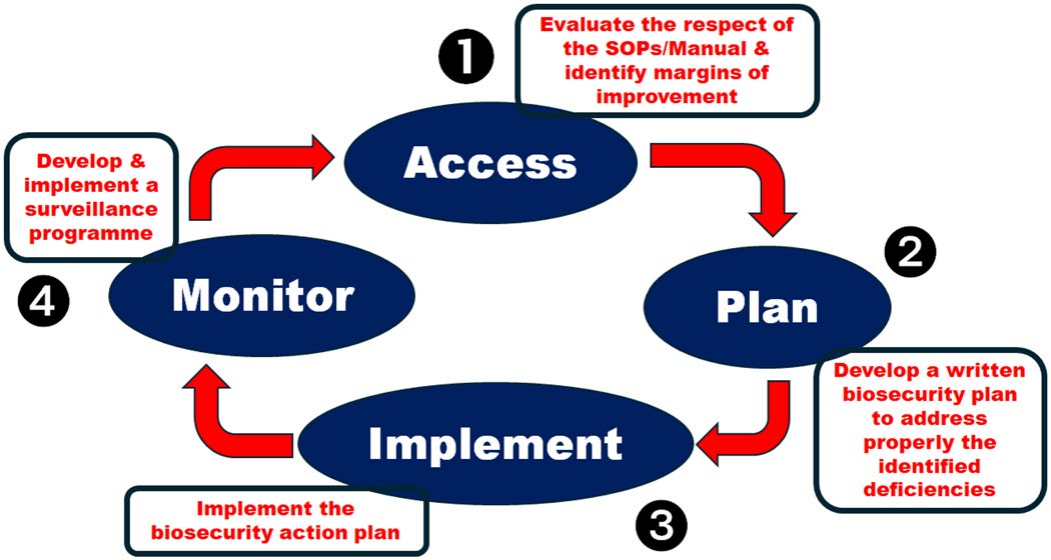
The virtuous circle for implementing and monitoring biosecurity standard operating policies and procedures.

The purpose of an audit is, first, to evaluate (❶ ‘access’) the effectiveness of compliance with the SOPs (manual) and to identify areas for improvement. The second step is to develop a written biosecurity plan to properly address the identified deficiencies (❷ ‘plan’). The third step is to implement the biosecurity action plan with an appropriate timeframe (❸ ‘implement’). Finally, a surveillance programme is developed and implemented to validate the effectiveness of the action plan (❹ ‘monitor’) (Figure 7).

**Figure 8.**
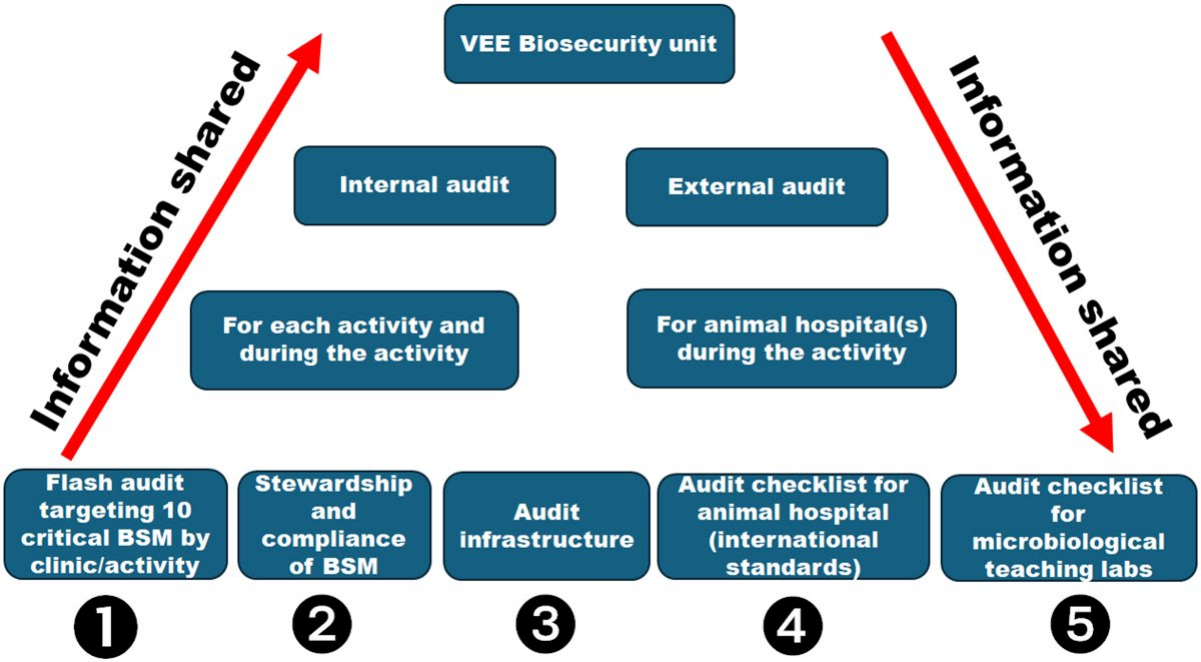
Organisation of audits related to VEE biosecurity SOP. *Legend.* BSM, biosecurity measures; ❶❷❸❹❺, five complementary audit tools.

Audits to assess the compliance with SOPs are performed regularly using different complementary tools (Figure 8).

**Figure 9.**
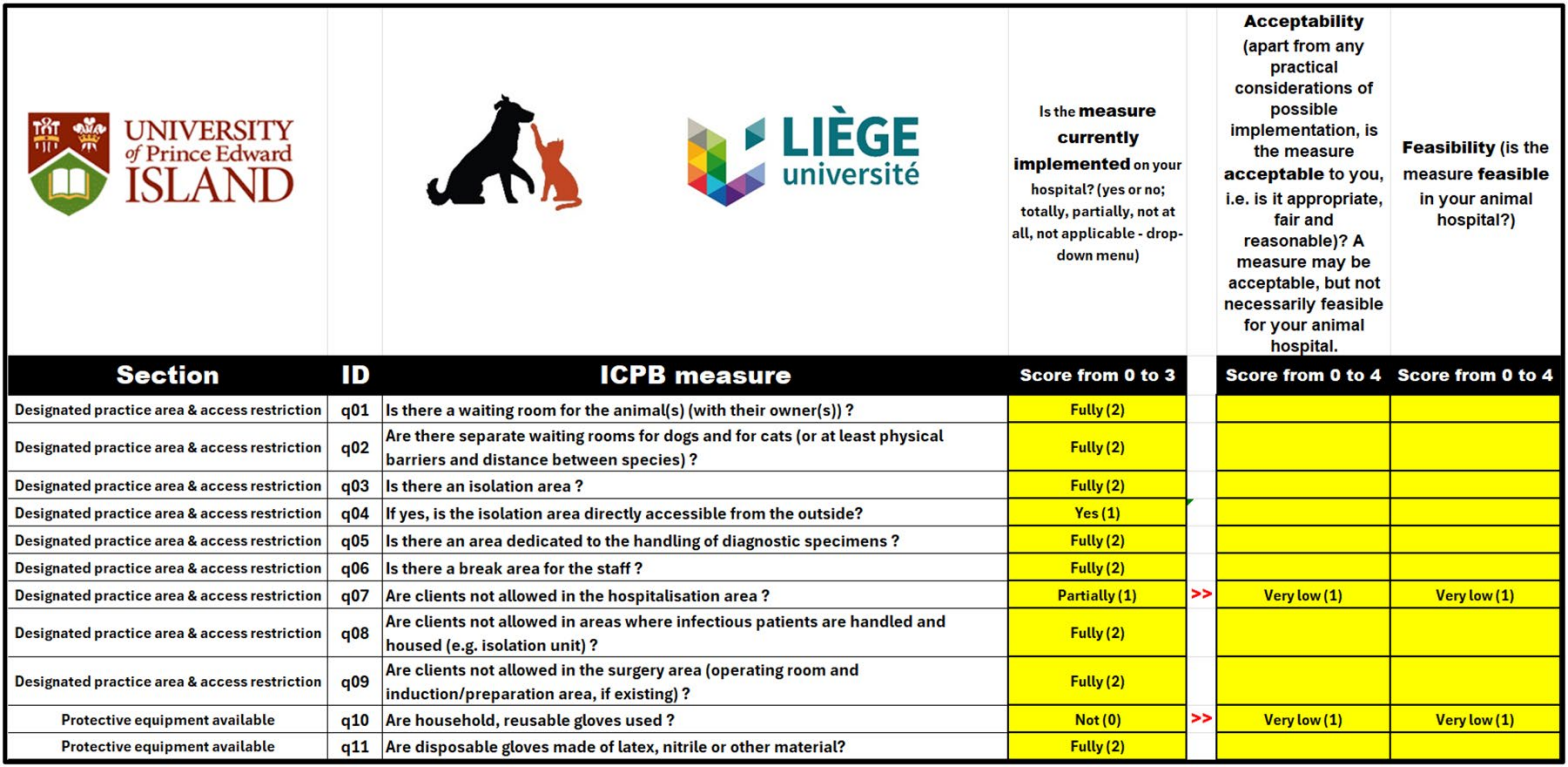
Example of audit for an animal hospital according to international standards (e.g. small animals), based on the recommendations of the American animal Hospital Association (adapted from Stull et al. 2018).

**Figure 10.**
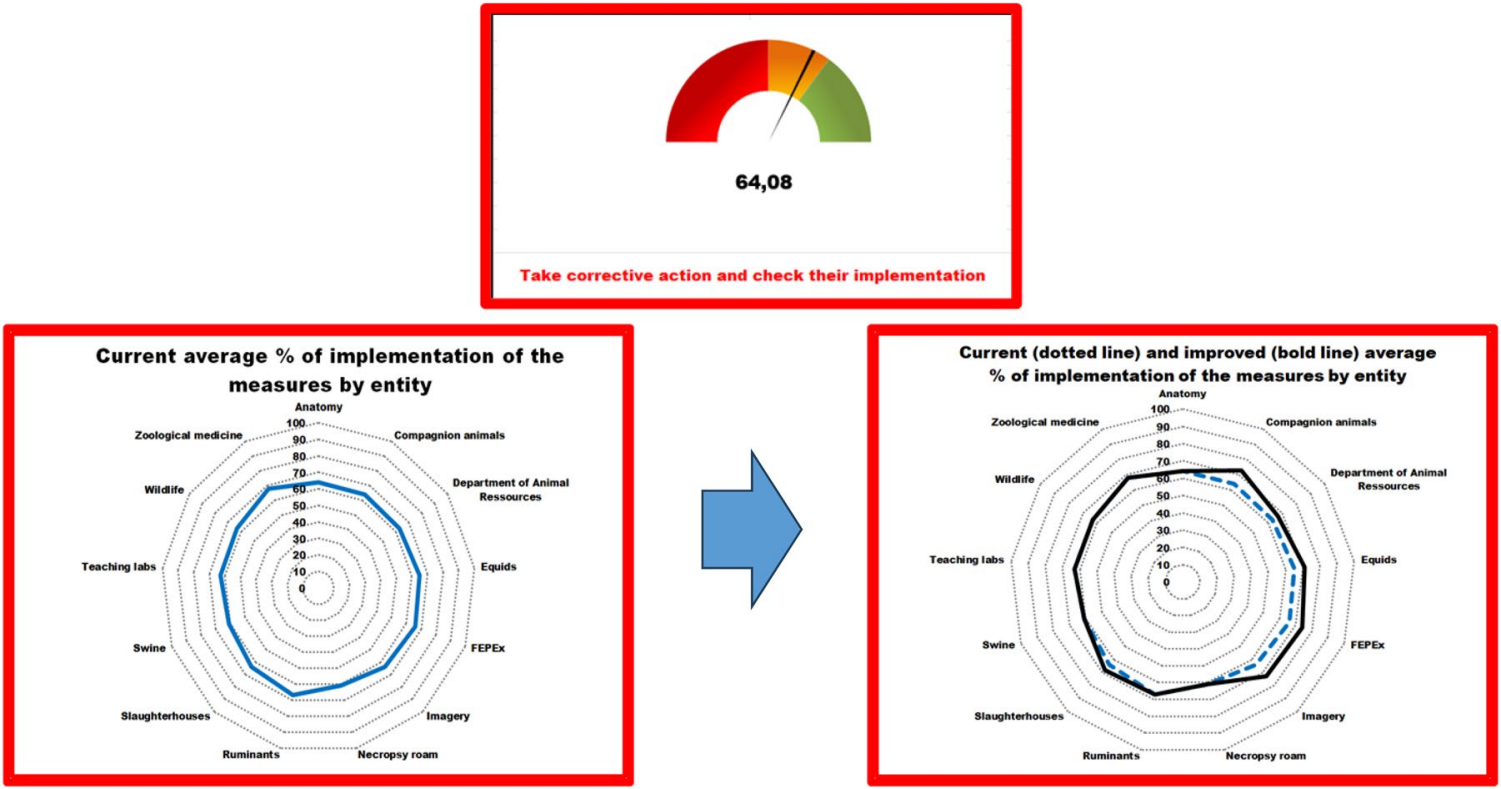
Dashboard of all clinics and activities inside the veterinary education establishment, using flash audits targeting ten critical points per clinic or activity. *Legend.* Above, a global barometer for the veterinary education establishment; Left, percentage of implementation of the measures per clinic or activity at the time of the audit; Right, percentage of implementation of the measures per clinic or activity after implementation of corrective action(s).

**Figure 11.**
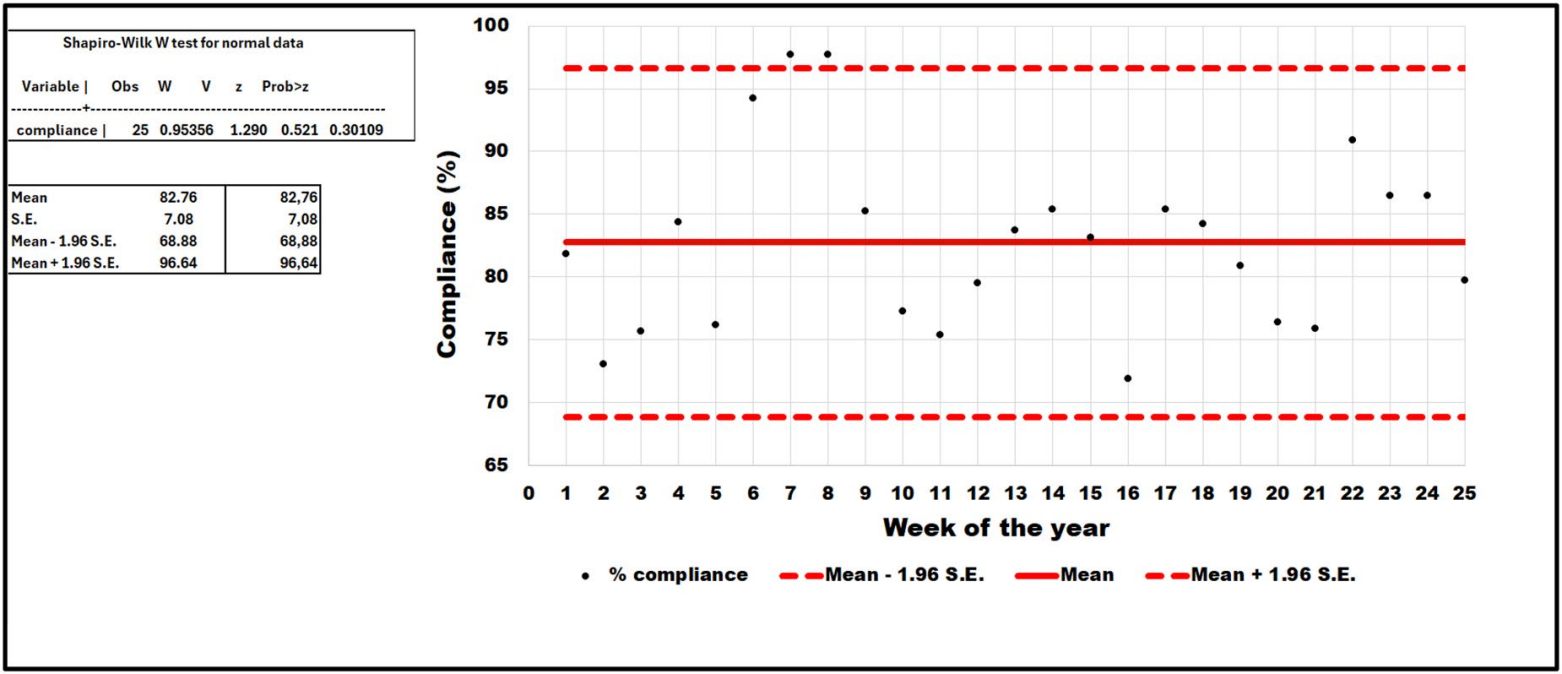
Trend analysis of weekly compliance rates with biosecurity measures in a clinic of ruminants. *Legend.* The weekly percentage of compliance with biosecurity measures follows a normal distribution (Shapiro-Wilk test; p-value = 0.30). The mean percentage of compliance +/- 1.96 standard error (S.E.) is plotted on the figure, corresponding to a 95% confidence interval. This allows identifying outliers, such as weeks 7 and 8, which correspond to exceptionally high compliance rates in the clinic.

- **Audits for veterinary hospital:** a specific audit form for veterinary hospitals has been adapted to European VEE using the 2018 Infection Control, Prevention, and Biosecurity Guidelines from the AAHA (Stull et al. 2018). Results are presented as a barometer after compiling scores and as a spider diagram, allowing rapid identification of problem areas. If a score is insufficient, recommended measures are automatically suggested based on their acceptability and feasibility, displaying the potential impact of each measure on the overall score (Figure 9).
- **Flash audits:** these sector-specific audits were developed based on the top 10 biosecurity measures that people must follow during their activities in the facilities (see Section “Implementation and auditing of biosecurity standard operating policies and procedures”) (Figure 10).
- **Stewardship:** some students or other dedicated people (e.g. technicians) are assigned to conduct, supervise or manage weekly biosecurity in the clinic or activity. For example, the clinic of ruminants has developed a checklist that includes 89 questions. In case of deficiencies, students or technicians propose corrective actions to ensure the proper functioning of the clinic during the week. This stewardship offers students and/or technicians a sense of responsibility for biosecurity (Figure 11).
- **Infrastructure audits**: these are carried out by the executive committee of the VEE biosecurity unit in preparation for EAEVE visits. These audits are unannounced inspections of infrastructures in facilities hosting teaching activities with biological risks. They help identify deficiencies of infrastructures and support requests for financial resources to overcome them (e.g. replacing tables and stools in anatomy dissection rooms, installing sinks in the large animal clinic, etc.). The audit framework includes identifying problems, developing an action plan to address them, tracking the status of each action, assigning responsibility and setting deadlines for completion.
- **Specific audit for microbiology teaching laboratories**: This audit was developed using the FAO Laboratory Mapping Tool (Mouillé et al., 2018) and further improved with elements from the checklist included in the document entitled ‘Medical biology analysis laboratories: assessment and prevention of infectious risks’ (INRS, 2024).

### Reporting a biosecurity issue

Reporting an issue can be done in several ways, in addition to the observations made during audits. For example, *via* above-mentioned biosecurity website, users can submit a biosecurity issue. The information is then immediately shared with all members of the VEE biosecurity unit *via* a specific email address, enabling a coordinated action.

In May 2023, the VEE implemented an online system for reporting adverse events affecting people directly or indirectly, regardless of their nature. This tool allows the VEE security officers and the biosecurity logistician to collect reports from ULiège staff members and students regarding incidents or accidents occurring in the VEE. Its purpose is to identify high-risk areas or activities and propose preventive actions, as well as adapt infrastructures and procedures when necessary. The system is easy (*via* a smartphone application) and quick to use, encouraging its broad use. Each report includes five key elements: the location and time of the event, people involved, a description of the event and any photo that contextualises it. According to the International Labour Organisation (ILO), an accident is an unexpected and unplanned event arising out of or in connection with work that results in personal injury, disease, or death, whereas an incident is any unplanned event that disrupts normal operations, with or without injury or damage. Accidents are therefore a subset of incidents where harm or injury occurs (ILO, 2015). The purpose of this reporting system is to detect warning signs (anticipation) before an accident occurs, so that preventing measures can be taken. As an example, during two years of use, 80 adverse events related to biosecurity in the faculty of veterinary medicine at the University of Liège (Belgium) were entered into the reporting system. Of these, 56 involved animals. The animal species involved were mentioned in 51 events: 14 events involved a horse (e.g. kicking, crushing against a wall, crushing of the foot, twisting of the hand during restraint), 37 involving mostly cats and sometimes dogs (31 bites or scratches), 7 punctures from used needles, and 4 cuts (e.g. scalpel blades, tin cans). As a result, an awareness campaign was launched in the small animal clinic to reduce the bites and scratches, by strengthening student training in animal restraint provided by veterinary assistants. Correct horse handling was also reaffirmed during clinic training.

## Discussion

This section describes the best practices to develop an animal biosecurity framework inside a VEE, supported by scientific literature references. In this section we discuss also the place of the distance education and e-learning in veterinary medicine with a focus on animal biosecurity. Finally, we compare the animal biosecurity framework presented in relation to other works and we present some perspectives.

### Veterinary education establishment biosecurity unit

Biosecurity governance requires the identification and active involvement of all actors who may be affected by biosecurity issues or who can influence the efficacy of biosecurity (Reed and Curzon 2015). The establishment of biosecurity governance in a VEE first needs the creation of a specific consultative entity that brings together all key players of the VEE, including students, and assumes responsibility for biosecurity across all facilities (clinics, facilities, teaching laboratories). This entity must be created on a democratic basis and recognized by the VEE authority. In addition, according to scientific literature, the presence of specific actors and stakeholders significantly increases the planning of activities (Brody 2003). Regular meetings should be organized throughout the year to make appropriate decisions. An executive committee should be identified for daily operations, rapid assessment, and decision making. Regular feedback to the dean of the VEE is of prime importance, and all activities should be compiled in meeting reports and in an annual report to ensure transparency and information sharing.

### Standard operating policies and procedures

Standard operating policies and procedures are the keystone of biosecurity within a VEE. These SOPs must be developed for each clinic and activity involving biological risks, following the guidance of the VEE biosecurity unit, which should include students. All SOPs should be assembled into a Manual (handbook), updated every five years by the VEE biosecurity unit and made freely accessible online. Whenever an activity or infrastructure changes (e.g. creation of a new clinic), the corresponding SOP must be updated and communicated to all relevant stakeholders. A printed copy of the SOP should be available in each clinic or department. A pictogram with a QR code that links directly to the biosecurity website should be displayed in every activity location, allowing students and staff members to directly access the relevant SOP at any time.

### Biosecurity website

The development of a specific updated website on animal biosecurity is strongly recommended. This website should illustrate and operationalize existing SOPs and guide visitors through essential information (e.g. location of a specific clinic, requirements before hospitalisation, rules to follow during hospitalisation, precautions after a patient discharge). Furthermore, the website should be a centre of knowledge for biosecurity, sharing up-to-date information (e.g. summaries after each biosecurity day). A system of issue reporting should also be included on the website.

### Biosecurity learning

According to the WHO, FAO and WOAH, biosecurity is included in the competencies for One Health field epidemiology (COHFE 2024). Indeed, we recommend the integration of biosecurity in the curriculum of animal epidemiology with clear reference to biosecurity. Generally, in the VEE curriculum, the observation, handling, containment and care of animals are competencies for both preclinical (healthy animals) and clinical (sick animals) students. Biosecurity principles and SOPs are important to deliver to preclinical students, especially before moving to clinics and animal hospitals, to avoid any errors in biosecurity protocols that can impact the animal patient, staff, owner, and the student himself/herself (e.g. biting), and the environment. Training in biosecurity should enhance awareness about the transmission of diseases and target the main principles of biosecurity and quality assurance related to the SOPs. A mix between face-to-face and eLearning activities is possible, with regular formative evaluation. In clinical degree, more attention should be devoted to biosecurity during different activities (clinics, facilities, teaching lab) and specific paraclinics on biosecurity with the objective of students performing by themselves a biosecurity audit in a facility under the supervision of a coach to support and stimulate initiatives. For veterinary practitioners (especially those that supervise students), continuing education on biosecurity is needed, targeting their needs and expectations (Saegerman et al. 2024).

### Annual biosecurity day

The themes addressed during annual biosecurity days raise awareness among staff, supervisors, students, veterinary practitioners and authorities about important issues, promote reflection and collective action on specific topics (developing a community of biosecurity practices), and mobilize resources for causes. We recommend that the topic of each annual biosecurity day be defined by participants of the previous year. Biosecurity days should be practical, to promote an inclusive, active and participative approach (Saegerman et al. 2004). Moreover, this participation increases engagement and responsibility of the community regarding biosecurity at the VEE level.

### Implementation and audit of biosecurity

Implementation of biosecurity SOPs by students is stimulated by communication sharing (Moya et al. 2025), stewardship (Kwik et al. 2003) and is facilitated when biosecurity is based on evidence (Xu and Yuan 2019). The conduct of an audit is a key element in learning about the implementation of biosecurity SOPs and in identifying areas for improvement. This improvement is a continuous process (virtuous circle) and must consider the fact that people are replaced over time. We recommend the implementation of several types of audits and the involvement of students and staff members in the process. In this way, they are more empowered and engaged in biosecurity of clinics, facilities, and teaching labs. Audit digitalization allows an easier integration and summarization of information at the clinic, facility, department, activity or VEE level. Improving biosecurity is progressive and requires vision and support (Box 2).

Box 2. Progressive pathway to improve biosecurity.

**Table:** 

A clear vision provided by the biosecurity team and supported by the Dean Step-by-step approach Acknowledgement is better than criticism Good ideas for improvement do not come solely form the ‘boss’ Regular assessment based on a co-constructed grid and form Uses of biosecurity day and practical training as levers for change

### Reporting biosecurity incidents

The purpose of an incident reporting system must be to prevent further accidents. In several domains (e.g. aviation, radiology), the detection and reporting of incidents help to prevent accidents (Nicholson and Tait 2002; Sarvananthan et al. 2021). Digitalization of the reporting system should be an incentive to obtain more high-quality data (e.g. development of an app, use of a QR code). The system should also be sufficiently sensitive and biosecurity-specific. Indeed, the definition should evolve over time to reach a good compromise between sensitivity and specificity of the system. Importantly, a maximum number of incidents should be reported and analysed. We recommend the regular data analysis by the VEE biosecurity unit to identify activities at risk, the reasons for incidents and areas for improvement (management options to prevent accidents). Upon responding to the notifier, the app administrators remind the importance of following the mandatory procedure in force in the Institution in the event of an accident (for insurance purposes and follow-up by the internal and external, i.e. occupational health doctor, services for prevention and protection at work). Key strategies include proper training, adherence to safety protocols, use of PPE, and robust reporting and analysis of incidents.

### Place of the distance education and e-learning in veterinary medicine with a focus on animal biosecurity

The Council on Education (COE) of the American Veterinary Medical Association (AVMA) is recognized by the United States Department of Education as the accrediting agency for programs that lead to professional degrees in veterinary medicine. Following the COVID-19 experience in distance education and e-learning development (DE) and in response to feedback from stakeholders indicated that online learning at veterinary colleges should enhance, not replace, in-person learning, recently the COE approved new policies on DE. Indeed, veterinary programs must (AVMA, 2025b):

- Remain predominantly residential, ensuring technology complements, not substitutes, in-person education;
- Establish clear oversight and approval processes for DE courses (excluding guest lectures or emergency sessions);
- Invest in technology, faculty training, and instructional design to support high-quality DE;
- Regularly assess student performance and outcomes across both in-person and online courses;
- Maintain at least 85% of the preclinical curriculum and 50% of direct instruction in a face-to-face format;
- Ensure regular and substantive faculty-student interaction in online courses.

According to recent studies that scan European needs and expectations related to livestock biosecurity training by using the World Café method, for veterinary practitioners, it was considered that a mixed approach, including a theoretical (e.g. basic principles) and a practical part where people are invited to audit a farm, create a biosecurity plan that includes acceptability and feasibility of the additional biosecurity measures to propose and a follow-up report, were the most important features of veterinary training (Saegerman et al. 2024; Iatrou et al. 2025; Saegerman et al. 2025).

Currently, few preliminary studies in veterinary/animal sciences suggest online and in-person learning often yield similar performance outcomes (knowledge, skills) with some studies showing online potentially better for specific tasks, but with trade-offs like reduced social interaction and motivation/focus challenges online (e.g. Hontoir et al. 2023; Aslım et al. 2024; Morris et al. 2024); blended learning often emerges as superior, highlighting that well-designed, engaging digital content can match traditional methods (e.g. Shandilya and Kaur 2025), though lab/hands-on components require careful online adaptation (e.g. May et al. 2023). For biosecurity, we recommend larger multicentric, multidisciplinary, critical, evidence-based studies and meta-analyses to compare DE and face-to-face education (advantages/disadvantages/performance related to outcomes) in order to gain more supportive reference to maintain or refine the current COE decision. However, in case of pandemic lockdown, disaster or armed conflict, DE remains always an alternative (Reimers and Schleicher 2020).

### Comparison of the animal biosecurity framework presented in relation to other works and perspectives

The proposal methodology was developed since 2010 with at the beginning few available supporting references (Linton et al. 1987; Quinn and Markey, 2000; Royal College of Pathologists, 2002; OIE, 2006; Wheeler and Dallap Schar, 2008; Petersen et al. 2008; College of Veterinary Medicine and Biomedical Sciences 2008). From these references only the biosecurity SOP of the College of Veterinary Medicine and Biomedical Sciences (Colorado State University) applied a comparable methodology (Morley et al. 2011). Since 2010, the ULiege biosecurity SOPs have been tested, improved using novel literature references and updated each 5-year period. Currently, these SOPs have been used worldwide and have become guidelines for EAEVE. Further development should be the production of DE materials and to becoming a recognised knowledge centre on animal biosecurity in VEE that delivers expertise, assessment, teaching education materials, stages for trainees and residents, and tools.

Other perspectives include the development, use and sharing of real time digital audit tools (AAHA 2019; Saegerman et al. 2024), the development of virtual farm tours (Tilli et al. 2024), serious games and simulation platforms (Koliba et al. 2022; Smith et al. 2023; Messina et al. 2023), the inclusion of artificial intelligence to assess compliance with biosecurity (Lima-Campêlo et al. 2024), edition of book chapters (e.g. Sarrazin et al. 2018; Saegerman and Humblet 2018), development of a community of practices (Militzer et al. 2023), of a COST-Action dedicated to VEE biosecurity, of practical training schools in animal biosecurity (Saegerman and Humblet 2022), and a Massive Open Online Courses (MOOCs) on VEE biosecurity (Millett and Shang, 2024). However, all these initiatives need to find funding or support to move from contemplation to action.

Innovative ideas are emerging in biosecurity, including the use of radio-frequency-identification-based (RFID) technology to monitor compliance with boot and hand sanitization when entering and exiting a barn (Racicot et al. 2022), adenosine triphosphate (ATP) luminometry to assess the equipment cleanliness (Buczinski et al. 2022), virtual fencing to facilitate livestock management (Schillings et al. 2024) and facial recognition systems for controlled entry in a specific area, using deep learning and a cloud-based service (Loume et al. 2022).

## Conclusion

The creation of a VEE biosecurity unit, the development and implementation of biosecurity SOPs, the number of visits of the dedicated biosecurity website, the number of individuals trained, and the number of biosecurity audits performed are all key factors contributing to the biosecurity in VEEs. Participation of students in these processes allows understanding, appropriation and empowerment regarding animal biosecurity both within and beyond the VEE. Equally important is the promotion of evidence-based biosecurity practices.
